# Harmful Algal Blooms in Aquaculture Systems in Ngerengere Catchment, Morogoro, Tanzania: Stakeholder’s Experiences and Perception

**DOI:** 10.3390/ijerph18094928

**Published:** 2021-05-06

**Authors:** Offoro Neema Kimambo, Jabulani Ray Gumbo, Hector Chikoore, Titus Alfred Makudali Msagati

**Affiliations:** 1 Department of Geography & Environmental Studies, Solomon Mahlangu College of Science & Education, Sokoine University of Agriculture, Morogoro 67115, Tanzania; 2 Department of Ecology & Resource Management, School of Environmental Sciences, University of Venda, Thohoyandou 0950, South Africa; 3 Department of Hydrology and Water Resources, School of Environmental Sciences, University of Venda, Thohoyandou 0950, South Africa; jabulani.gumbo@univen.ac.za; 4 Unit for Environmental Science and Management, North-West University, Vanserdbijlpark 1900, South Africa; 32945280@nwu.ac.za; 5 College of Science, Engineering & Technology, University of South Africa, Johannesburg 1709, South Africa; msagatam@unisa.ac.za

**Keywords:** cyanobacteria, harmful algal blooms, stakeholder perceptions, water pollutions, aquaculture systems, Ngerengere catchment, Morogoro

## Abstract

The aquaculture sector has experienced fast growth as a result of livelihood diversification initiatives among small-scale farmers in Tanzania. Regrettably, the dynamics of harmful algal blooms (HABs) have been overlooked despite the noticeable forcing of climate variability, the interaction between social-economic activities, and domestic water supply reservoirs. This study aimed at surveying the occurrence, experiences, and perceptions of HABs in aquaculture systems from stakeholders in the Ngerengere catchment, Morogoro, Tanzania. A cross-sectional survey focus group discussion (FDG), key informant interviews, and anecdotal observation were adopted. A convenient and purposive sample population was drawn from pond owners, registered water users, and government officials in the catchment. For data analysis, descriptive statistics and constant comparison were performed. Most respondents (95%) were able to recognize the image of blooms displayed. Approximately 70% of the respondents agreed that water quality has deteriorated over time, and blooms occur during the dry season. Further, 60% of the respondents agreed that water pollution is a serious problem attributed to sources other than industrial discharge. There was no consensus regarding the health impacts associated with HABs. Raising awareness on HABs is of paramount importance as it will provide the basis for the development of HABs management framework and health risk assessment.

## 1. Introduction

Harmful algae are photosynthetic and microscopic bacteria that are naturally occurring in marine and freshwater ecosystems [1]. Cyanobacteria produce secondary metabolites (toxins), for example, microcystins, cylindrospermopsin, anatoxins, and saxitoxins, which are harmful to fish, domestic animals, and humans [2]. In most cases, harmful algal blooms (HABs) and cyanobacterial harmful algal blooms (CyanoHABs) have been used interchangeably to describe cyanobacteria species that tend to produce toxins, alter the food web, or produce hypoxia. A current global discussion is on the dynamics of cyanobacterial HABs in freshwaters with a changing environment and climate change [3]. Brooks et al. [4] suggest that the magnitude, frequency, and duration of HABs are poorly understood and also HABs have received inadequate attention [5] and that this is a global problem [6].

Small-scale fish farmers (traditional fisheries) have been and will continue to be the most vulnerable to HABs besides challenges on startup capital, operating resources, and poor farming practices [7]. East Africa is an economical water scarcity area [8], and apart from that, there has been resistance in financing aquaculture projects [9]. Environmental factors such as land degradation, pollution (point and non-point sources), climate and hydrological variability, habitat loss (conversion of wetlands into fishponds) also add pressure on small-scale fisheries and the whole ecosystem. HABs in the Ngerengere catchment situated in Morogoro, The United Republic of Tanzania, are not well documented. A survey in the Wami Ruvu basin found water pollution to be a significant problem and recommended increasing awareness and ecotoxicological studies [10]. The implication can be evidenced in a social economic profile of the Morogoro region which itemized 10 most common causes of morbidity, including diarrhea and skin diseases, which are also symptoms of some cyanotoxins exposure [11]. A comparative study of microbial community in three clusters (pristine, urban, and agriculture) identified *Cylindrospermopsis* which is among the harmful algal-forming Cyanobacteria species [12].

In Tanzania, fish production statistics stands at 1% for aquaculture, 14% for marine, and 85% for inland (lakes, rivers, etc.) waters [13]. According to the Ministry of Livestock and Fisheries Development [13], Tanzania mainland aquaculture fish farmers increased from 3347 to 17,511 between the years 2000 and 2013 with a corresponding increase in number ponds from 4000 to 19930 and landed production from 200 to 2989.5 tons [14]. Thus, social-economic factors are critical for the intensification of fish farming in the region, which is mainly due to emphasis on extension education of farming practices to the practicing farmers [15] and technological improvements [9]. In a survey of the Morogoro region, despite efforts, aquaculture is still in a nascent stage and intensively operated by small-scale farmers [16].

The harmful impacts of environmental changes, such as climate change and weather variability on HABs dynamics and attendant effect on food security has received less attention, especially in the pursuit of sustainable development goals and the 2030 Agenda [4]. To our knowledge, no study has yielded findings on the awareness of HABs from water users in the catchment. Only a few cases have shown their concern about water quality standards for fishing and environment that are yet to be established [17,18]. Unfortunately, farmers cannot access water quality parameters to inform their decisions; rather, they rely on qualitative measures such as water source, color changes, effects on fish, and inability to locate markets [19]. For example, health effects are also perceived to be connected to low water quality by farmers [20]. With algal blooms, there are already reported cases [21]. A study in Ukerewe, an island in Lake Victoria, evidenced the health impacts of cyanobacteria-contaminated drinking water in the area [22]. One way to overcome the problem is to assess the occurrence, timing, and awareness of HABs and their health effects in the catchment through an interdisciplinary collaboration [23]. For this study, it was of interest to investigate how HABs are perceived (occurrence, extent, and timing) by water users, government workers (water sector), and small-scale fish farmers in Ngerengere catchment. Therefore, the objective of the current study is to survey the occurrence and perception of harmful algae in aquaculture systems in the Ngerengere catchment, a sub-catchment of the main Wami Ruvu basin located in the Morogoro region, the United Republic of Tanzania.

## 2. Materials and Methods

### 2.1. Study Area Description

**Figure 1:**
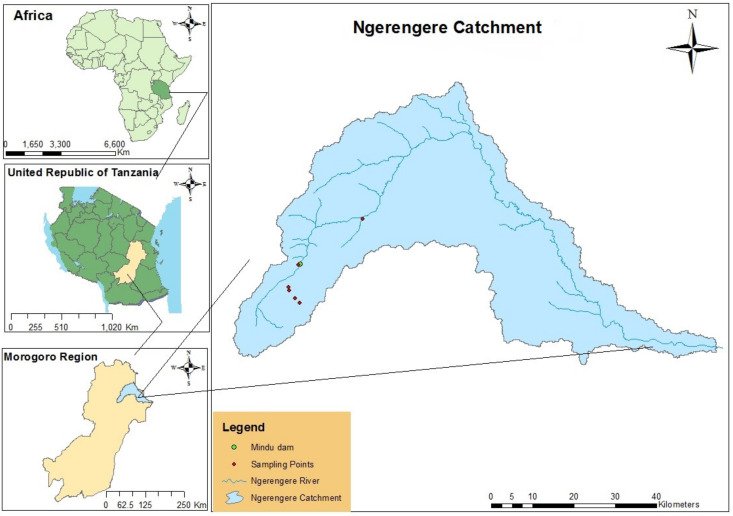
Study area map (adapted with permission from Kimambo et al. [33]).

The Ngerengere catchment is the sub-catchment of the main Wami Ruvu basin, located in the Morogoro region, Tanzania, within longitudes and latitudes of 37°32′ E 6°51′ S, 38°09′ E 6°69′ S, 37°38′ E 7°09′ S, and 38°38′ E 7°05′ S, respectively (Figure 1). It covers approximately an area of 2780 km^2^ and is characterized by a tropical climate [24]. Mindu Dam is the primary source of water and freshwater fishery supplies in the urban and peri-urban of Morogoro [25]. However, erosion and sedimentations due to human activities are more prominent challenges [26] and these have continuously reduced the depth of the Dam and Ngerengere River [27]. Water quality status and trends in the catchment have also been studied with an indication that there is significant pollution contributed by agriculture, domestic, and industrial activities [12,28,29,30,31]. Furthermore, recent work on chlorophyll-a and variations in climate and hydrology has highlighted some possible causes of HABs in the catchment [32].

### 2.2. Study Design

The current study consisted of mixed methods (observation, focus group discussion, questionnaires, and key informant interviews). The approach has been found to be helpful, especially in research that lacks a body of research [20,34], the present study being the case. A pre-tested questionnaire coded both in English & Kiswahili languages was uploaded into SurveyCTO an open data kit (ODK)-based (available at https://www.surveycto.com/index.html, accessed on 5 October 2017), and android-assisted application to gather the required information. The questionnaire had three sections designed to collect social-demographic information and knowledge (which consists of how HABs appear, causes, threats, experiences of the respondents in the study area on pollution and water quality, HABs, and their management or control measures). Before embarking on field surveys, the questionnaire was pretested and validated in an area close to the study area. This was an expert-driven pretesting, that tested the flow, order, timing and language navigation (English to Kiswahili). It was done by comparing answers from one pretest and another. The aim was to identify problems with questions or response options in the survey.

The sample size conveniently and purposively consisted of 31 respondents from small-scale fish farms, officials of the Morogoro Urban Water Supply Authority (MORUWASA), Wami Ruvu Basin Office (WRBO), and the registered water users (the list of water users was obtained from WRBO). Since the study consulted experienced personnel in the catchment, the sample size was theoretical as in Gholami et al. [35] (i.e., “10–30 expert opinion for a decision making group would be an effective”), who assessed environmental risk assessment of harmful algal blooms. Additionally, five key informants were from Morogoro districts and the deputy director of Tanzania Fishery Institute was contacted. Along with that, two focus group discussions with five participants each and one meeting with MORUWASSA officials were held. During field campaigns (October 2017, February 2018, May 2018, and August 2018, which aimed to conduct water sampling and in situ findings), several reservoirs were visited for the visual identification/observation of blooms. Since pond size has a significant impact on production and management but also the quality and size of the fish [9], we focused on fish farmers whose pond sizes measure at least 100 m^2^ on the basis of the previous studies [15]. Some livelihood activities (agriculture and fishing) in upstream settlements and erosion have been observed in the catchment [36]. Mindu Dam (a reservoir for domestic water supply) was included in an attempt to capture such interactions and their possible causes.

### 2.3. Socio-Economic Status

The Ngerengere catchment has an estimated population of over one million people [36]. A recent survey [37] on the Wami Ruvu basin noted industries, agriculture, mining, and settlement as the critical socio-economic and livelihood activities. The survey further elucidated that pollution (point and non-point sources), increased demand for water uses in agriculture, and increased urban population triggers water-scarce conditions at times in the catchment. A project jointly led by Global Water for Sustainability, Florida International University (GLOWSFIU) and Wami Ruvu basin office on water quality [29] noted conflicts between downstream and upstream water users on water quality in the basin. In history, the Morogoro region was considered an ordinary town in Tanzania and possibly in a more considerable part of Africa [38], due to the location along the major transport routes (roads and train to other mainland towns), the status of being one of the selected towns for concentrated urban development, the closeness to the business city Dar es Salaam, and the area of potential for agricultural development. In Morogoro, the number of fish farms have recently increased mainly due to diversification of livelihoods and local markets [39,40,41].

### 2.4. Data Analysis

Data were downloaded from the computer server provided by the SurveyCTO in Microsoft Excel format and transferred for further analysis. Since the study adopted digital data collection, the service provider (SurveyCTO) offers features of quality control from forms/questionnaire design, data collection, monitoring and transferring from server to an intended statistical analysis package. Images of blooms, mat, and foam-like from field observations were presented as captured. The study adopted a content analysis [34] approach for analyzing the qualitative information. Jeffreys’s Amazing Statistics Program (JASP) computer software (version 0.9.0 of 2018) was used to produce descriptive plots and Chi-Square tests statistics for drawing inferential statements. The description of the significance and interpretation of the results in all the tables (VS-MPR) was adopted from [42]. The choice of JASP considered it is potentials over other tools, for example, it is a simple, attractive graphical user interface, freely available and open-source computer program but also as demonstrated in the previous studies [43,44,45].

### 2.5. Ethical Consideration

An ethical clearance certificate with reference number SES/17/ERM/09/2006 was issued by the Research Ethics Committee (REC) in the Directorate of Research & Innovation of the University of Venda, Limpopo, South Africa.

## 3. Results

### 3.1. Respondents General and Experiences

This subsection presents information about gender, marital status, education, and number of years the respondents stayed in the study area.

**Figure 2:**
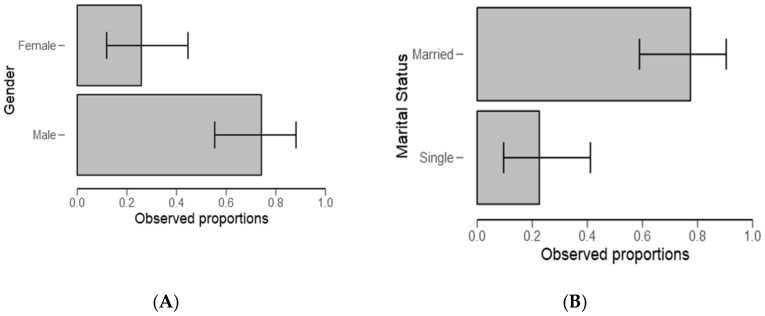
Gender (**A**) and marital status (**B**) descriptive plots for all the respondents.

In the current study, significantly (respectively, *p* = 0.007; *p*= 0.002, Table 1), most respondents were male and married (see the proportion in Figure 2A,B).

**Figure 3:**
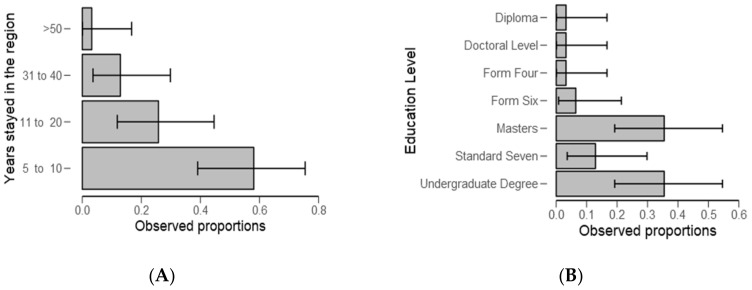
Descriptive plots for number of years respondents stayed in the study area (**A**) and education level (**B**).

**Figure 4:**
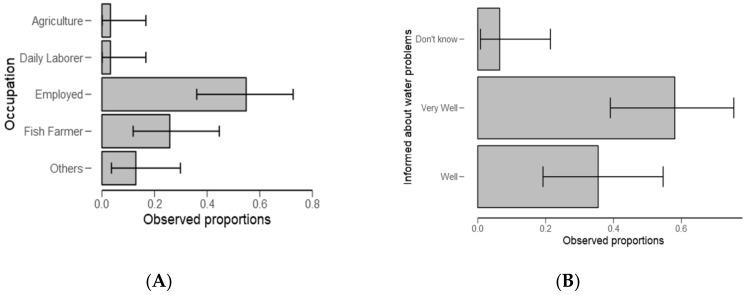
Descriptive plots for occupation (**A**) and how informed the respondents are about water problems in the Ngerengere catchment (**B**).

From Table 2, significantly (*p* < 0.01) of the examined categories, respondents (59%) stayed in the study area for about 5 to 10 years (Figure 3A) and 37% had high levels (*p* < 0.05) of education (Figure 3B and Table 2). Furthermore, a higher number of respondents (59%) were employed (Figure 4A and Table 3) (*p* < 0.05) and they were “very well” informed about water problems in the study area (Figure 4B) (*p* = 0.002, Table 3).

### 3.2. Water Quality and Algal Bloom Formation

**Figure 5:**
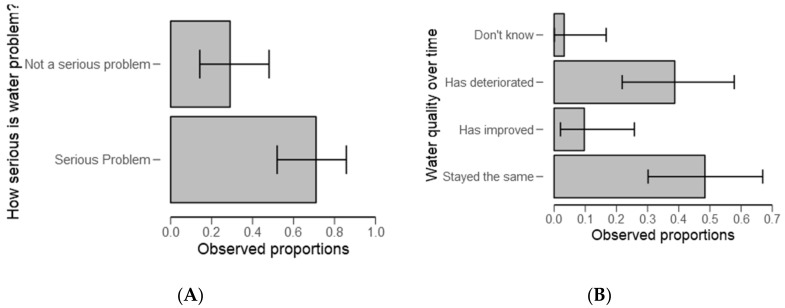
Descriptive plots of water problems (**A**) and quality over time (**B**).

Asked about whether water problems in the region are serious or not serious, respondents (70%) collectively agreed that water problems in the catchment are “serious” (Figure 5A). The findings also suggest that 49% of the respondents noted no change in water quality, with 40% who affirmed that water quality has deteriorated over time (*p* < 0.05) (Figure 5B and Table 4).

**Figure 6:**
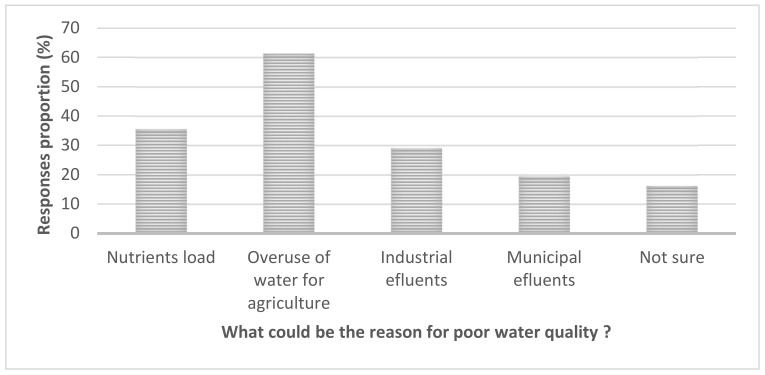
Ranks (response in %) for the reasons of poor water quality in the study area.

In Figure 6 we describe the results from multiple response options on “what could be the reasons for poor water quality”. Here overuse of water for agriculture ranked higher (60%) than other responses.

**Figure 7:**
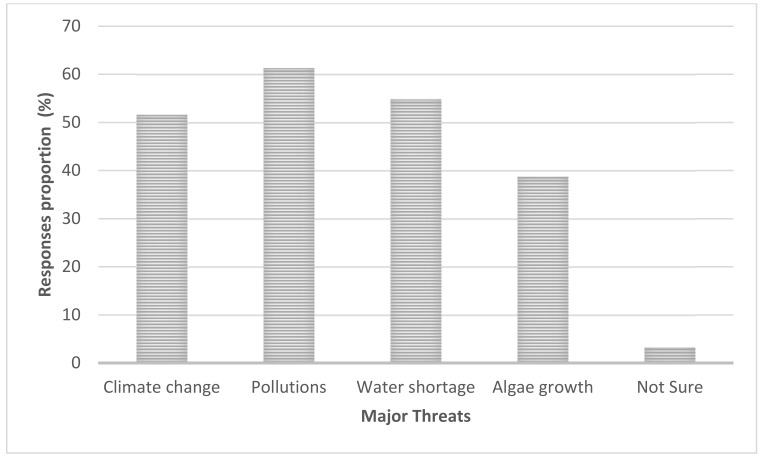
Ranks for the major threats as perceived by the respondents.

When asked about the major threats, pollution ranked high (60%) followed by water shortage and climate change, which altogether accounts for 50% of the respondents (Figure 7).

**Figure 8:**
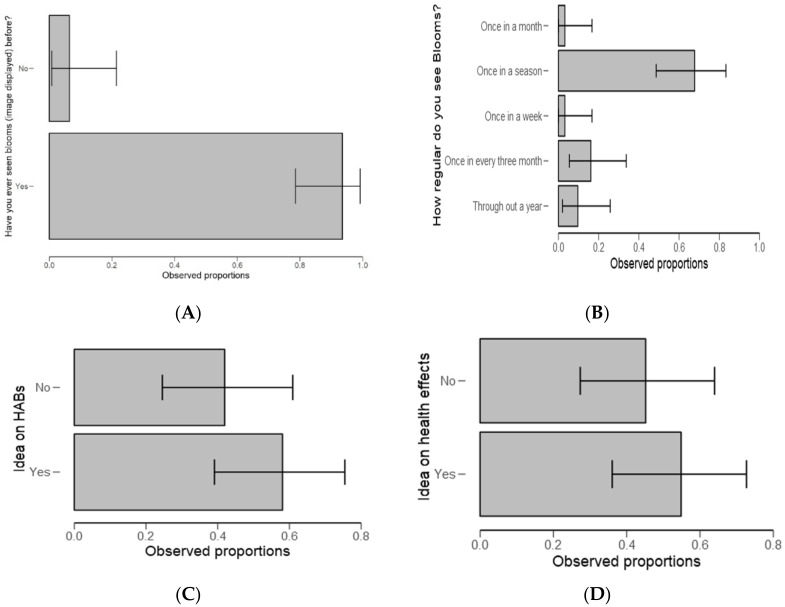
Descriptive plots for harmful algal blooms (HABs) recognition (**A**), how regular do blooms occur (**B**), idea/aware on HABs (**C**) and the idea/aware of HABs health effects (**D**).

The test statistics revealed that respondents were highly aware (>95%) of algal blooms feature (Figure 8A). Herein, the algal bloom image was displayed to the respondent for recognition during the survey (Table 5). It was further observed that respondents collectively agreed that blooms usually occur once in a season and during the dry season (Figure 8B).

When asked about any idea on HABs in the ponds/dam or river (Figure 8C) and any idea about health effects associated with algal blooms (Figure 8D), there was no significant difference between the groups (*p* = 0.369 and *p* = 0.590, respectively).

**Figure 9:**
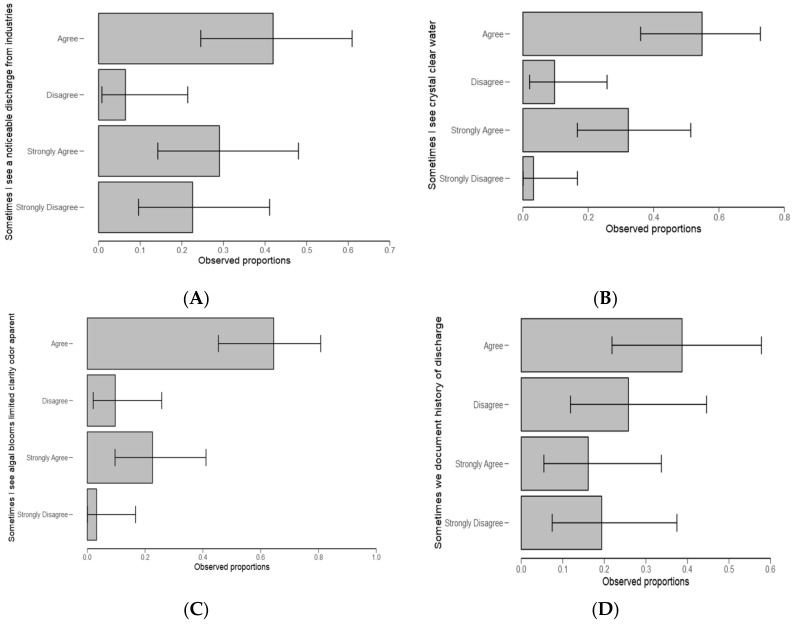
Aspects tested in recognition of HABs formation and their course in water. (**A**): Sometimes I see a noticeable discharge from industries; (**B**): Sometimes I see clear crystal water(**C**): Sometimes I see algal blooms with limited clarity and odour apparent (**D**): Sometimes we document history of discharge.

**Figure 10:**
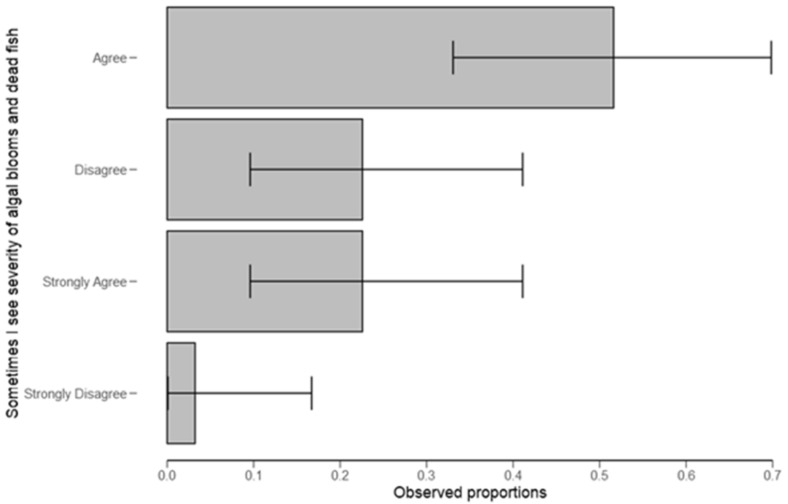
Ranks on whether sometimes an object sees the severity of algal blooms and dead fish.

From Figure 9A, respondents collectively agreed that sometimes there is a noticeable discharge from the industries, and sometimes they see crystal-clear water (Figure 9B). It was further found that collectively, respondents agreed sometimes they see algal blooms limited with clarity odor apparently (Figure 9C). Otherwise, there was no significant difference between the groups (Figure 9D) when asked about documenting discharge history (Table 6). Moreover, most respondents (52%) agreed to have seen the severity of algal blooms (Figure 10) and dead fish (Table 6).

**Figure 11:**
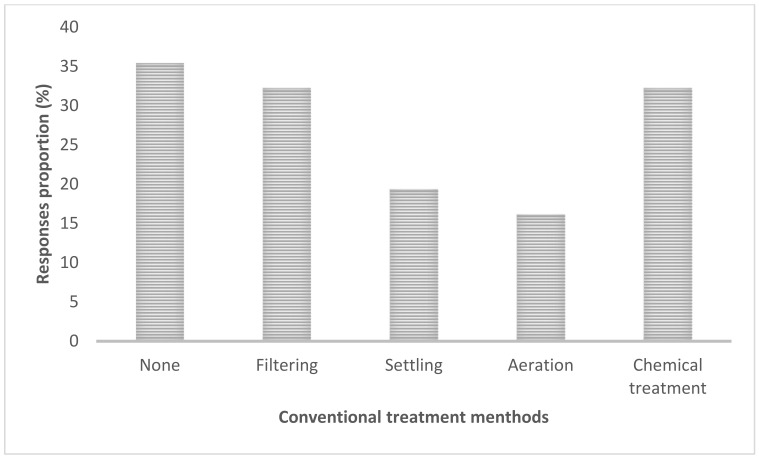
The conventional methods for HABs control.

When the respondents were asked about measures in place to control measures of HABS, 35% noted that no treatment method is applied (Figure 11), followed by filtering and a chemical treatment (both scored same proportion of 32%).

### 3.3. Field Observation

In the present study, several images/plates of blooms were taken from different reservoirs during field excursion, and that is presented as captured.

## 4. Discussion

### 4.1. Demographic Features

Most ponds activities, including monitoring and fishing, are performed by men (Table 1 and Figure 2). The results provide evidence to the prevailing point of view that gender inequalities are common in the fishery sector and consistent with the previous studies [46,47]. Regarding experience, most respondents have stayed in the study area for a period ranging from 5 to 10 years (Table 2). This may not have affected study findings because the current study design targeted people who regularly intermingle with water users, authorities, small-scale fish farmers, and experts. The findings are in line with the previous study wherein the same were also observed in Kilombero by Kangalawe [48], which is a district neighboring the study area, and in the same fish farming livelihood activity. These findings raise a concern about gender participation in fish farming, and in the context of our study, men might be more exposed to a risks associated with the presence of harmful algal blooms.

### 4.2. Perceived Water Quality Over Time

In the present study, respondent were very well informed about water problems (Table 3) but also out of the tested groups (serious or not serious) respondents (70%) collectively agreed that water problems in the catchment are “serious” (Figure 5A), and this dovetails nicely with the previous surveys (e.g., [37]). Regarding water quality and algal bloom formation, the National Water Sector Development Strategy of 2006–2015 stresses the links between water quality and fisheries but also the impact of pollution on fisheries [49]. In this report, water-use conflicts between downstream and upstream communities were evident. A similar pattern of results was obtained, for example, during the experts’ interview, water quality was eyed as an issue of concern (Moshi, M, personal communication, 7 August 2018). Furthermore, 49% of the respondents noted that there was no change in water quality over the years (Figure 5B), with 40% who affirmed that water quality has deteriorated over time (*p* < 0.05) (Table 4). These results demonstrate the high degree of uncertainty over water quality changes. This triangulation implies a need for interventions in the catchment and to refine study by getting more audience.

Figure 6 shows that overuse of water for agriculture (mostly paddy and maize as per observation and the national survey [50]) scored high, followed by nutrients loads and industrial effluents. This was confirmed during the key informant interviews, which revealed that controlling agricultural activities upstream of Mindu dam is lacking (Angumbwike, N. personal communication, 29 August 2018). Regarding the possible threats in the study area, from Figure 7, for example, increase in pollution ranked the highest (60%) followed by water shortage and climate change, which altogether accounted for 50% of the respondents, and these were broadly in line with the observation of World Bank [51]. Others also cited algal growth as a problem, although the rating was lower than other options, but this could be attributed to low and lack of awareness on HABs dynamics.

In testing on knowledge on algal blooms, the results demonstrate two things. Firstly, respondents were highly aware (Figure 8A and Table 5) of how algal blooms appear (when a photo of algal bloom were displayed for recognition). Secondly, respondents collectively agreed that blooms usually occur once in a season with most of them referring to the dry season (Figure 8B); likewise for the focus group discussions (FGD), and the interviews. The results indicates the best timing for studying HABs occurrence and mobility, however, HABs can form any time of the year as in [52]. The results corroborate the findings of [53] on tropical cyanobacteria blooms and the verbatim comments from the respondents in clarifying the season as a factor in algae blooming: “green algae blooms in Mindu Dam proliferate mostly during the dry season”. Therefore, the responses inform the best timing for the planning of pre- and post-management/control of HABs.

### 4.3. Perceptions of HABs on Health Effects

It is widely accepted that some species of harmful algal blooms can cause skin irritations [54,55]. During a focus group discussion with the fish farmers, it was revealed sometimes that they (the farmers) had experienced the same. For example, one interviewee pointed out that they must have soap with them and change clothes because they normally feel skin irritation just after fishing (Raphael, I., personal communication, 10 August 2018).

From the interviews, we speculate that the irritation of skin might be associated with algal bloom effects or it could be other factors. This implies that there is a need for further investigation and implementation of public health awareness rising on the effects of HABs apparently. It is with regret that guidelines are yet to be developed in Tanzania. To verify the concern in the previous studies [17,18], in a key informant interview, there was a claim that current guidelines and standards for the management of algal blooms are yet to be in place (Maly, R., personal communication, 7 August 2018). These primary findings are consistent with the previous study, which shows that the issue of HABs is not well addressed in policies and guidelines [18]. Similarly, a recent review noted that there are still questions that need to be answered, especially on policies and ecosystem change with climate change and population increase [46].

During the key informant interviews, some noted the policy gap and agreed that conservation training and awareness-raising are considered as an immediate solution for managing harmful algal blooms. Verbatim comments commended the current study in the catchment; for example, “this project will help us identify problems of water quality in the catchment” (Angumbwike, N., personal communication, 29 August 2018). These observations are in line with the study by van der Heijde et al. [56].

This also agreed with most respondents’ verbatim comments that there is a need to raise awareness but also proposing an intervention strategy. These findings pose concerns about policy and practices on the fishery and the environment. When respondents asked about any idea on HABs in the ponds/dam or river (Figure 8C) and any idea about health effects associated with algal blooms (Figure 8D), there was no significant difference among groups (Yes/No). The results also highlight that little is known about HABs and as well as health effects associated with algal blooms. Furthermore, during the interviews and the FGD, the same uncertainty featured; for example, a statement made by one of the interviewees that “some species of algae could be toxic, but not sure.” (Dunia Mlanzi, Personal Communication 6 August 2018).

These findings are similar to observations that have been reported in the previous surveys and most important in a developed world whereby 60% of fishermen in Southern Louisiana did not know what HABs mean [57]. Extension services seem to be a key constraint for Tanzania farmers as the issue features in many reports [56,58]. These findings stress concerns about programs to increase awareness that need to be addressed either through training and more from extensions services.

In order to verify the respondent’s concerns on the link between water quality problem and any observed ecological responses, using Likert scale questions (Figure 9A, Table 6), respondents collectively agreed that sometimes there is a noticeable discharge from the industries. Respondents agreed that they sometimes see crystal-clear water (Figure 9B). They also agreed sometimes they see algal blooms limited with clarity odor apparently (Figure 9C). On the other hand, there was no significant difference among groups (Figure 9D) when asked about documenting discharge history (Table 6). Moreover, most respondents (52%) agreed to have seen the severity of algal blooms (Figure 10) and dead fish (Table 6). When comparing our results to those of earlier studies, similar observations were made; for example, fishes dying because of polluted water as in Niang [37]. This may alter or improve aspects of the monitoring of HABs in the catchment.

### 4.4. Harmful Algal Blooms Management and Control

Regarding the conventional control measures of HABs, 35% of respondents said no treatment method is applied (Figure 11), followed by filtering and chemical treatment (both scoring 30%). Herein the design (i.e., asking multiple-choice questions) utilized was meant to probe more reactions from the respondents. As a part of management, it was interesting to note during the focus group discussion and interviews that farmers use hand palm mimicking Secchi disk (for Secchi disk depth) technique for monitoring the turbidity in their fishponds. Water is added into the pond if they cannot see the palm of their hand, baseline being the Elbow. During FDG farmers brought to the table an issue of reduced yield, specifically fish sizes being small as compared to large lakes fishes. As an intervention, farmers at times make use of chalk lime before introducing fingerlings or just after fishing: “Chalked lime is applied (Chokaa in Kiswahili) to the fishpond before introducing Fingerlets and just after harvesting” (Mlegu, D., personal communication, 10 August 2018).

This dovetail well with the principle understanding that some other fishponds management techniques, for example, the use of lime have been tested for sterilization, nutrient enrichment, and for regulating pH changes [59].

**Figure 12:**
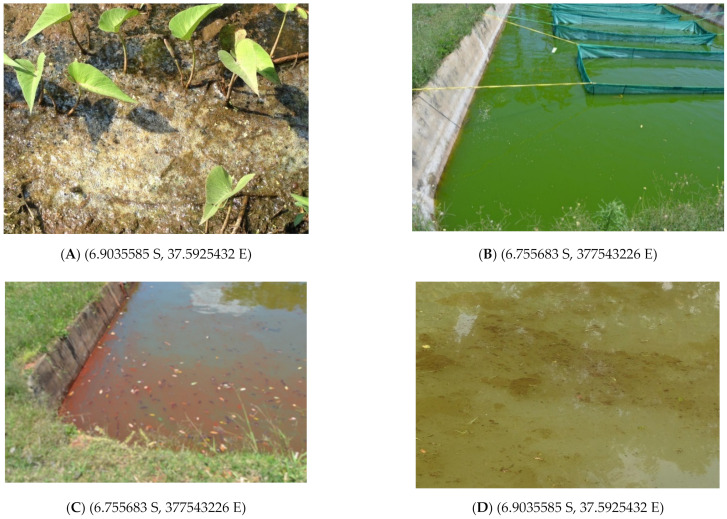
Visible foam-like algae as observed at Konga, Kidangawa (**A**), Greenish colorations as observed at Kingolwira fishponds (**B**); next to it is red algae (**C**), and finally mat-like algae as observed at Konga, Kidangawa (**D**). Specific location, i.e., latitude and longitudes, in the brackets (photos by the author during the survey).

In the present study, field images/observations agree with respondents’ comments and key informant interviews. For example, the key informant interview pointed that blooming occur mostly during the dry season (July, August, September, October, and November) (Angumbwike, N, personal communication, 29 August 2018). The difference in blooming (i.e., mat, bloom and foam-like) and the difference in colors in (Figure 12A–D) requires more studies in the catchment.

## 5. Conclusions

This study aimed to investigate the occurrence and perception of harmful algal blooms in the Ngerengere catchment in Morogoro, Tanzania. The findings confirm that respondents are very well informed about the problems of water quality and the reasons for the cause, such as overuse of water for agriculture, and nutrients. Respondents were able to identify algal blooms when an image of bloom displayed to them and they collectively agreed that algal bloom proliferates more during the dry seasons (June to September and sometimes in January to February). That tallied with the anecdotal observations which showed the occurrences of algal blooms of all forms (bloom, mat, and foam-like) and that some had a red hue. On the other hand, there was no consensus regarding the health effects associated with HABs. In addition, respondents collectively agreed that they sometimes see the severity of algal blooms and dead fish. The findings challenge policymakers, technical specialists (e.g., medical practitioners), and researchers together to address problems associated with algal blooms, specifically HABs. The findings provide a basis for the development of HABs management framework (i.e., education and extension programs, identification, monitoring, and control). While the present study provides useful insight about HABs in Ngerengere catchment, the implication may be specific to the study area. Since the sample size was small and specific to stakeholders around the Upper catchment of Ngerengere, the results may reflect only the people of urban Morogoro. Future researches should consider monitoring environmental conditions, toxic strains identification, and their mobility. There is a need to obtain wider scale results that are representative of the whole country.

## Figures and Tables

**Table 1:** Gender and marital status multinomial test.

Parameter	Chi Squire (χ^2^)	Degree of Freedom (df)	(*p*-Value) *p*	Vovk-Sellke Maximum *p* Ration (VS-MPR ^a^)
Gender	7.258	1	0.007	10.522
Marital Status	9.323	1	0.002	26.684

^a^ Vovk-Sellke Maximum *p*-Ratio: Based on the *p*-value, the maximum possible odds in favor of H_1_ over H_0_ equals 1/(-e p log(p)) for *p* ≤ 0.37 [42]. Here, respectively, *p*-values of 0.007 and 0.002 are only 10.522 and 26.984 times more likely favoring an alternative hypothesis than the null hypothesis (more clarification can be obtained at http://www.shinyapps.org/apps/vs-mpr, accessed on 26 October 2018).

**Table 2:** Years stayed in the region and education level multinomial test.

Parameter	Chi Squire (χ^2^)	Degree of Freedom (df)	(*p*-Value) *p*	Vovk-Sellke Maximum *p* Ration (VS-MPRa)
How many years have you stayed in the region?	21.258	3	<0.001	425.916
Education level (H_0_ (a))	28.839	6	<0.001	584.901

**Table 3:** Occupation and how the subject is informed about the water problem multinomial test.

Parameter	Chi Squire (χ^2^)	Degree of Freedom (df)	(*p*-Value) *p*	Vovk-Sellke Maximum *p* Ration (VS-MPRa)
Occupation	28.839	4	<0.001	3735.115
How well are you informed about the problems facing water sources in the region?	12.452	2	0.002	29.877

**Table 4:** Water problem and quality over time multinomial test.

Parameter	Chi Squire (χ^2^)	Degree of Freedom (df)	(*p*-Value) *p*	Vovk-Sellke Maximum *p* Ration (VS-MPRa)
How serious about the water-related problem?	5.452	1	0.020	4.782
How are the changes in water quality for the time you have been in the region?	17.903	3	<0.001	103.970

**Table 5:** Respondents understanding of HABs multinomial test.

Parameter	Chi Squire (χ^2^)	Degree of Freedom (df)	(*p*-Value) *p*	Vovk-Sellke Maximum *p* Ration (VS-MPRa)
Have you ever seen blooms (image of bloom displayed for recognition) before?	23.516	1	<0.001	21,834.894
How regularly do you see blooms?	45.935	4	<0.001	7.318 × 10^6^
Do you have any idea on HABs in river/ponds/dam/reservoir?	0.806	1	0.369	1.000
Any idea about health effects associated with algal blooms?	0.290	1	0.590	1.000

**Table 6:** Multinomial test for several aspects tested for recognition of blooms.

Parameter	Chi Squire (χ^2^)	Degree of Freedom (df)	(*p*-Value) *p*	Vovk-Sellke Maximum *p* Ration (VS-MPRa)
Sometimes I see a noticeable discharge from industries	8.097	3	0.044	2.675
Sometimes we document a history of discharge	3.710	3	0.295	1.022
Sometimes I see a crystal-clear water	20.484	3	<0.001	306.378
Sometimes I see algal blooms with limited clarity and odor apparent	28.226	3	<0.001	8941.006
Sometimes I see the severity of algal blooms with one or more of the following, massive floating scum, strong foul odor and dead fish	14.806	3	0.002	29.726

## Data Availability

Data used (as guided by the ethical clearance) to support the findings of this study are available from the corresponding author upon request.
